# Matrix Stiffness Contributes to Cancer Progression by Regulating Transcription Factors

**DOI:** 10.3390/cancers14041049

**Published:** 2022-02-18

**Authors:** Seiichiro Ishihara, Hisashi Haga

**Affiliations:** Department of Advanced Transdisciplinary Sciences, Faculty of Advanced Life Science, Hokkaido University, N10-W8, Kita-ku, Sapporo 060-0810, Japan; haga@sci.hokudai.ac.jp

**Keywords:** cancer, stiffness, extracellular matrix, mechanotransduction, transcription factors, cancer associated fibroblasts, YAP/TAZ, collagen, contraction, crosslinking

## Abstract

**Simple Summary:**

Matrix stiffness is recognized as a critical factor in cancer progression. Recent studies have shown that matrix stiffening is caused by the accumulation, contraction, and crosslinking of the extracellular matrix by cancer and stromal cells. Cancer and stromal cells respond to matrix stiffness, which determines the phenotypes of these cells. In addition, matrix stiffness activates and/or inactivates specific transcription factors in cancer and stromal cells to regulate cancer progression. In this review, we discuss the mechanisms of cancer stiffening and progression that are regulated by transcription factors responding to matrix stiffness.

**Abstract:**

Matrix stiffness is critical for the progression of various types of cancers. In solid cancers such as mammary and pancreatic cancers, tumors often contain abnormally stiff tissues, mainly caused by stiff extracellular matrices due to accumulation, contraction, and crosslinking. Stiff extracellular matrices trigger mechanotransduction, the conversion of mechanical cues such as stiffness of the matrix to biochemical signaling in the cells, and as a result determine the cellular phenotypes of cancer and stromal cells in tumors. Transcription factors are key molecules for these processes, as they respond to matrix stiffness and are crucial for cellular behaviors. The Yes-associated protein (YAP)/transcriptional coactivator with PDZ-binding motif (TAZ) is one of the most studied transcription factors that is regulated by matrix stiffness. The YAP/TAZ are activated by a stiff matrix and promotes malignant phenotypes in cancer and stromal cells, including cancer-associated fibroblasts. In addition, other transcription factors such as β-catenin and nuclear factor kappa B (NF-κB) also play key roles in mechanotransduction in cancer tissues. In this review, the mechanisms of stiffening cancer tissues are introduced, and the transcription factors regulated by matrix stiffness in cancer and stromal cells and their roles in cancer progression are shown.

## 1. Introduction

Cancer tissue is often recognized as solid and desmoplastic. Cancer tissues are stiffer than normal or adjacent tissues in various types of organs. For instance, mammary cancer tissue is stiffer (~4 kPa) than normal mammary tissue (~0.2 kPa) [1]. Liver stiffness below 6 kPa is recognized as normal tissue, whereas stiffness values over 8–12 kPa are designated as a disease state, such as fibrosis and cirrhosis, which could trigger hepatocellular carcinoma (HCC) [2]. The stiffness of normal healthy pancreatic tissue is approximately 1–3 kPa [3,4], while that of pancreatic cancer tissue is over 6 kPa [4]. For lung tissues, it has been suggested that the stiffness of lung solid tumors (20–30 kPa) is stiffer than that of normal lung parenchyma (0.5–5 kPa) [5]. High-grade serous ovarian cancers (HGSOC) show different stiffnesses depending on their subtype. Mesenchymal HGSOC, a much more aggressive subtype, displays stiffer tissues than non-mesenchymal HGSOC and less aggressive subtypes [6]. Gliomas are stiffer (~1 kPa) than non-malignant gliosis (0.1 kPa) [7] and highly malignant glioma tissues (~10 kPa) are much stiffer than low-grade or innocent glioma tissues (~3 kPa) [8]. In bladder cancer patients, recurrent cancer stiffness is ~13 kPa, whereas newly diagnosed cancer stiffness is ~8 kPa, and adjacent normal tissue stiffness is ~3 kPa [9]. Stiffness values were measured in specific ways such as elastography or atomic force microscopy. Therefore, the comparison of actual values between the studies is not appropriate; however, these studies indicate that the tissue stiffness of solid tumors is higher than that of corresponding normal tissues.

## 2. Mechanism of Cancer Stiffening

**Figure 1:**
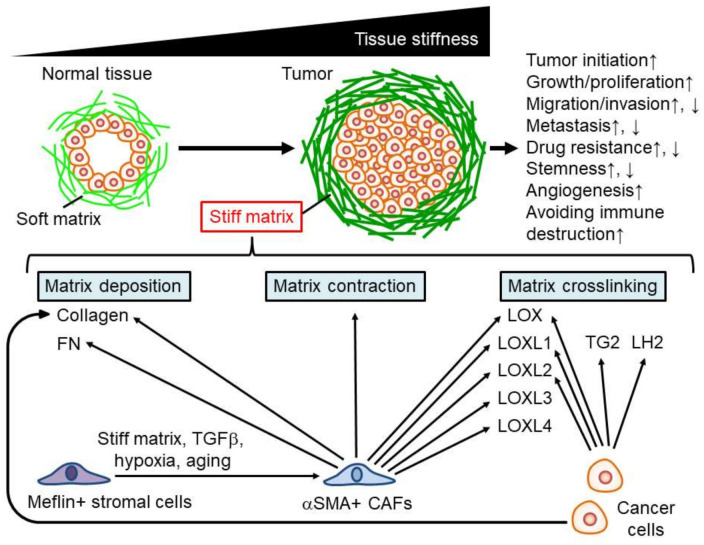
Mechanisms of cancer stiffening and contribution of matrix stiffness to cancer progression. ↑: upregulation, ↓: downregulation.

Previous studies have reported that tissue stiffening in solid tumors is regulated by cancer and stromal cells in tumor tissues. There are three major causes of cancer stiffening: (1) matrix deposition, (2) matrix contraction, and (3) matrix crosslinking (Figure 1). Cancer cells and stromal cells contribute to these events and determine the stiffness of cancer tissue.

### 2.1. Matrix Deposition

Increasing the extracellular matrix (e.g., collagen) density elevates matrix stiffness [10]. Mesenchymal stromal cells in tumors often show a high expression of alpha-smooth muscle actin (aSMA), which is a typical myofibroblast marker. These cells are known as cancer-associated fibroblasts (CAFs). Recently, there have been several names for this type of cell (e.g., cancer-promoting CAFs (pCAFs), myofibroblasts, and activated fibroblasts). CAFs secrete stiffness-promoting matrix components such as collagen and fibronectin (FN) [11,12,13]. Cancer cells also secrete matrix proteins such as collagen and enhance the stiffness of tumor tissues [14], suggesting that both CAFs and cancer cells are responsible for tumor tissue stiffening via matrix deposition.

### 2.2. Matrix Contraction

Extracellular matrices are remodeled by the contraction of surrounding cells in the tumor microenvironment. CAFs induce matrix contraction, followed by matrix stiffening in vitro [15]. Src homology 3 protein interacting with NCK, 90 kDa (SPIN90), is downregulated in mammary cancer stroma, increasing microtubule acetylation and as a result, promotes the transition of stromal cells to CAFs, generating high contraction, even on soft stroma mimicking early-stage cancer tissues [16]. Therefore, matrix remodeling for stiffening by CAF contraction may occur in the early stages of cancer progression.

### 2.3. Matrix Crosslinking

Matrix crosslinking is critical for stiffening cancer tissue. One of the major molecules contributing to matrix crosslinking is the lysyl oxidase family proteins, including lysyl oxidase (LOX), lysyl oxidase-like 1 (LOXL1), lysyl oxidase-like 2 (LOXL2), lysyl oxidase like 3 (LOXL3), and lysyl oxidase like 4 (LOXL4). Collagen crosslinking by LOX increases tumor stiffening in mammary cancer tissues [17]. LOX also plays an important role in the stiffness of colorectal cancer, as LOX-overexpressing colorectal cancer cells increase collagen crosslinking and, as a result, stiffen tumor tissues [18]. In HCC, the expression and secretion of LOXL2, but not LOX, LOXL1, LOXL3, or LOXL4, promote tissue stiffening [19]. Another study showed that LOXL1 is secreted by lung cancer cells [20]. CAFs also express LOX family molecules (LOX, LOXL1, LOXL2, LOXL3, and LOXL4) [13], suggesting that secretion of these molecules from CAFs is critical for tissue stiffening. Another study reported that polarized M2 macrophages express LOXL2 [21], suggesting that not only cancer cells and CAFs, but also other stromal cells such as macrophages secrete proteins for matrix crosslinking.

Other secretions also contribute to the tissue stiffening of cancer cells. In lung tissue, the tumor stroma contains higher levels of hydroxylysine aldehyde-derived collagen cross-links (HLCCs) than that of normal lung tissues. Lysyl hydroxylase 2 (LH2) produced by lung cancer cells elevates the levels of HLCCs and enhances tissue stiffness in lung cancer [22]. Tissue transglutaminase (TG2), a Ca^2+^-dependent enzyme that crosslinks proteins, is highly expressed in pancreatic cancer cells and stiffens pancreatic tumor tissue by crosslinking collagens [23]. In addition, stromal cells, indicated as CAFs, in fibrotic tissues associated with HCCs reduce matrix metalloproteinase-9 (MMP-9) expression and elevate TIMP metallopeptidase inhibitor 1 (TIMP-1) expression [24]. These molecular switches may induce tissue stiffening through matrix accumulation.

### 2.4. Regulation of Stromal Cells for Tissue Stiffening

As we have shown, CAFs are critical for tissue stiffening in tumors. Normal fibroblasts generate a softer matrix than CAFs [25], indicating that fibroblasts in cancer tissues are reprogrammed by the surrounding tumor microenvironment. Indeed, previous studies have reported that the functions of CAFs are regulated by external stimuli in tumors. Hypoxia prevents contractions in CAFs, followed by decreased matrix stiffness via hypoxia-inducible factor 1-alpha (HIF1A) stabilization [26]. A high-fat diet enriches CAFs and increases tissue stiffness [11]. Furthermore, matrix stiffness is critical for the CAF phenotype, as a stiff matrix maintains CAF function [15] and triggers the differentiation to CAFs from mesenchymal stem cells [27]. Therefore, a positive feedback loop between tissue stiffening and CAFs may exist in the tumor microenvironment.

Recently, it was found that mesenchymal stromal cells expressing meflin, a glycosylphosphatidylinositol-anchored protein, showed tumor-suppressing effects by reducing LOX activity in the tumor stroma [28,29]. Various stimuli such as matrix stiffness, transforming growth factor beta (TGFβ), hypoxia, and aging prevent meflin expression in mesenchymal stromal cells and increase the expression of collagens [29,30,31]. These results indicate that during cancer progression, meflin expression decreases to promote the transition of stromal cells to CAFs for tissue stiffening in cancer.

## 3. Cancer Progression Regulated by Matrix Stiffness

Many cell types respond to matrix stiffness and change their phenotypes. For instance, a stiff matrix increases cell spreading by increasing stress fibers in fibroblasts [32]. Cell migration is also regulated by the matrix stiffness. The direction of single cell migration is guided by matrix stiffness, called “durotaxis”, as cells easily migrate across the boundary from a soft to a stiff matrix; on the other hand, fibroblasts find it hard to migrate toward a soft matrix from a stiff matrix [33]. Multicellular clusters of epithelial cells also show durotaxis, even though isolated single cells do not [34]. In addition, the soft matrix coordinates the collective migration of epithelial cells because the migrating direction of each cell in a colony is organized in one direction, whereas cells in a colony on a stiff matrix move in a random direction [35]. The migration speed of fibroblasts on a stiff matrix is higher than that on a soft matrix [36]. Furthermore, matrix stiffness determines the differentiation of stem cells: a soft matrix physically mimicking brain tissues induces differentiation to nerve cells, intermediate matrix stiffness similar to muscle tissues triggers differentiation to muscle cells, whereas a stiff matrix similar to bone tissues leads to differentiation of mesenchymal stem cells into bone cells [37]. As described above, various cell types respond to matrix stiffness and determine their phenotypes.

Cells in tumors, including cancer and stromal cells, also respond to matrix stiffness and regulate cancer progression by modulating their phenotypes (Figure 1). Various cellular phenomena contribute to cancer progression [38]. Abnormal cell proliferation in normal cells initiates tumorigenesis. Cancer cell proliferation is essential for tumor growth. Migration and invasion abilities are critical for destroying surrounding tissues. Metastasis, defined as the generation of secondary tumors in organs distant from the primary tumor, is crucial for increasing the difficulty of curing cancer. Drug resistance, cancer cell stemness, angiogenesis, and immune reactions are important for cancer progression. Here, we introduce the effects of matrix stiffness on these phenomena in cancer cells.

### 3.1. Cancer Initiation Regulated by Matrix Stiffness

Cancer initiation first occurs by abnormal cell proliferation—that is, the transition of a normal cell to a cancer cell. Previous studies have shown that matrix stiffness is critical for this event. Mammary epithelial cells in soft matrices in culture display normal epithelial tubulogenesis; however, in stiff matrices, they show abnormal morphology, similarly to tumors [39]. Another study reported that culturing epithelial cells on a stiff matrix mimicking tumor tissues elevated cytoskeletal tension, which perturbs tissue polarity, increases growth, and disrupts normal lumen formation, resembling tumor initiation [1]. Multicellular sheets of mammary epithelial cells on a stiff matrix also show high proliferative capacity, similar to tumor initiation, via escape from contact inhibition [40]. Another study showed that mammary cell spheroids on the matrix with dynamic transit of stiffness from a soft matrix to a stiff matrix represent morphological changes that lose epithelial characteristics and gain mesenchymal phenomena close to tumor morphology [41]. A stiff matrix induces a tumorigenic phenotype through changes in the chromatin state in a three-dimensional (3D) culture mammary cancer model [42].

Animal studies have also shown that high collagen density in mammary tissues enhances tumor incidence in a mouse model, suggesting that a stiff matrix caused by dense collagen tissues is a critical factor for tumor formation [43]. Indeed, increasing collagen density enhances matrix stiffness, which induces a malignant, tumor-like morphology, proliferation in mammary epithelial cells [10,44] and skin thickening with hyperproliferation [45]. Collagen crosslinking by LOX is also crucial for matrix stiffening and increases tumor incidence in a mouse model [17].

Matrix stiffening regulates tumor incidence along with other factors. Transforming growth factor beta (TGFβ) is known to have two opposite functions in cancer progression: tumor suppressor function by inhibiting proliferation and inducing apoptosis, and tumorigenic functions by inducing epithelial–mesenchymal transition (EMT) and increasing cell migration in cancer cells. In normal mammary gland cells and kidney epithelial cells on a soft matrix, TGFβ induces apoptosis; on the other hand, on a stiff matrix, TGFβ treatment results in EMT induction [46]. In addition, in mammary epithelial cells, increasing matrix stiffness promotes cancerous phenotypes, whereas these effects are abrogated by increasing basement-membrane ligands [47]. These studies indicate that matrix stiffness and other factors regulate tumor initiation in a coordinated manner.

As shown above, matrix stiffening increases the risk of cancer initiation. This suggests that tissue stiffening without cancer cells is critical for cancer progression. Indeed, mammographic studies have demonstrated that desmoplastic, stiff areas are present in the normal human breast tissue, and consist of a reduced number of adipocytes and an increased number of fibroblasts and fibrillar collagens [48]. The presence of these mammary desmoplastic and stiff tissues increases the risk of breast cancer. Therefore, in addition to the interaction between cancer and stromal cells, normal stiff tissues are critical for cancer progression.

### 3.2. Proliferation of Cancer Cells Regulated by Matrix Stiffness

To generate tumors, cancer cells proliferate and form clusters. In addition to normal cells, cancer cells also respond to matrix stiffness and regulate their proliferation. A stiff matrix enhances the proliferative ability of cancer cells in HCC [49], colorectal cancer [18,50], lung cancer [20,51], and pancreatic cancer [23]. Cancer cell proliferation is also supported by the indirect effects of matrix stiffness—that is, the stimulation of cancer cell proliferation by the surrounding stromal cells. Mesenchymal stem cells differentiated into CAFs on a stiff matrix secrete the soluble factor prosaposin, which promotes the proliferation of mammary cancer cells [27]. Additionally, a stiff matrix induces autophagy in stromal cells such as fibroblasts and stellate cells, which enhances the growth of adjacent cancer cells [52]. Therefore, a stiff matrix increases the proliferation of cancer cells through both direct and indirect effects.

### 3.3. Migration/Invasion of Cancer Cells Regulated by Matrix Stiffness

Cell migration (direct movement of the cells) and cell invasion (movement of the cells across compartment of the tissues) are important for cancer progression. The potential for the migration and invasion of cancer cells is a malignant phenomenon because cancer cells degrade and destroy surrounding tissues via these processes. In addition, migration and invasion are critical steps in many cases of metastasis, which is a severe situation in patients with cancer. Matrix stiffness is a key factor for migration and invasion. A stiff matrix results in a migrating or invasive phenotype in colorectal cancer cells [18], lung cancer cells [22], mammary epithelial/cancer cells [26,53,54], HCC cells [55,56], squamous cell carcinoma (SCC) cells [57], ovarian cancer cells [58], and osteosarcoma cells [59]. In specific cases, the invasion of cancer cells is promoted by EMT: the transition of epithelial non-migrating cells to mesenchymal migrating cells. Stiff matrix is reported to trigger EMT and, as a result, facilitate the invasion of cancer cells [53,54,59].

It has also been reported that two-dimensional (2D) cell migration without physical barriers on a 2D matrix and 3D cell invasion with matrix degradation or deformation in a 3D matrix are regulated differently depending on matrix stiffness. Previous studies have reported that cells on a stiff 2D matrix migrate more actively than those on a soft 2D matrix [36,55,58,59]. On the other hand, in a 3D matrix, mammary cancer cells show delayed invasion compared to cells in a soft matrix [60]. In addition, in a 3D matrix, the formation and functions of invadopodia for invading the 3D matrix are restricted by the stiff matrix [61]. Another study reported that moderate matrix stiffness promotes mammary cancer cell motility on a 2D matrix [62]. Therefore, it appears that a stiff matrix might positively regulate migration of cancer cells on a 2D matrix and negatively regulate their invasion in a 3D matrix. Another study reported that the invasion of cancer cells is also brought about by CAFs, which are stimulated by a stiff matrix [15], suggesting that the contribution of matrix stiffness to migration and invasion may occur in various ways, including the stimulation of cancer cells and stromal cells, dependent on the surrounding environment.

### 3.4. Metastasis Regulated by Matrix Stiffness

In many cases, metastasis is a lethal event in cancer patients. Metastasis is suggested to occur in multiple steps: the local invasion of cancer cells to surrounding tissues, the entry of cancer cells into blood or lymphatic vessels (intravasation), the transit of cancer cells through the vessels, the exit of cancer cells from the vessels to distant organs (extravasation), and the generation of secondary tumors by growing in the tissues [38]. Each step is critical for the success of metastasis in cancer.

Previous studies have indicated that tissue stiffness is crucial for metastasis. Increasing tissue stiffness in primary tumors enhances the metastatic potential of cancer cells in HCC [19,63], lung cancer [14,20,22], and mammary cancer [26,53,54,60,64,65]. Matrix stiffening affects not only cancer cells but also non-cancerous cells during metastasis. A stiff matrix modifies protein expression on the surface of endothelial cells and promotes metastasis by increasing the intravasation of cancer cells [66]. In addition, matrix stiffening regulates metabolic rewiring between cancer cells and CAFs in tumors and enhances the metastatic potential [67]. Furthermore, both primary tumor stiffness and matrix stiffness in distant tissues are critical for successful metastasis: a stiff matrix promotes metastasis [19,68]. Another study showed that enhancing tissue stiffness prevents metastasis by secreting prosaposin from CAFs [27], indicating that the stiff matrix negatively regulates metastasis by stimulating stromal cells. These studies suggest that the tissue stiffness of both primary tumors and metastatic regions positively and negatively regulates metastasis by stimulating cancer and stromal cells.

### 3.5. Drug Resistance Regulated by Matrix Stiffness

Drug resistance is one of the major reasons for difficulty in cancer therapy. Matrix stiffness has been reported to both positively and negatively contribute to drug resistance in cancer cells. For instance, patients with soft tumors in mammary cancer showed a better response to chemotherapy than those with stiff tumors [69]. The stiff matrix enhances drug resistance in Her2-amplified mammary cancer cells [70], HCC cells [55], and pancreatic cancer cells [71]. On the other hand, soft matrices have been reported to induce drug resistance in laryngeal squamous cell carcinoma cells [72], HCC cells [49,73], colorectal cancer cells [74], and triple-negative mammary cancer cells [75]. Gao et al. [55] investigated the resistance of HCC cells to sorafenib, whereas Schrader et al. [49] and Tian et al. [73] examined their resistance to cisplatin and 5-fluorouracil. Therefore, the response of matrix stiffness to drug resistance may be dependent on the cell strain and drug species. In addition, mammary cancer cells grown on intermediate stiff matrices showed the highest drug resistance [76]. Another study showed that mammary cancer cells on a matrix mimicking the stiffness of their host tumor have a high drug resistance potential [77], suggesting that changing the matrix stiffness may be a good method for cancer therapy with drug treatment. The culture dimensions are also critical for drug resistance. A previous study reported that on a 2D 400 Pa matrix, the incorporation of EdU, which is an indicator of cell proliferation, was approximately 50%, whereas in a 3D 400 Pa Matrigel, the EdU incorporation was approximately 20% following lapatinib treatment in HER2+ breast cancer cell line [70]. Another study showed that a stiff 2D matrix induces sorafenib and lapatinib resistance in breast cancer cells; in contrast, a stiff 3D matrix reduces drug resistance in the cells [78]. Therefore, drug resistance regulated by matrix stiffness is dependent on the culture dimension, suggesting that 3D culture models with different matrix stiffness to mimic tumor tissues in vivo are important for evaluating drug resistance in cancer cells with several future applications.

### 3.6. Stemness Regulated by Matrix Stiffness

Cancer stem cells were originally defined as cells operationally through their ability to efficiently seed new tumors upon inoculation into recipient host mice and represent higher drug resistance [38]. Therefore, the effects of matrix stiffness on the stemness of cancer cells and drug resistance are similar in some cases. Indeed, a stiff matrix induces stemness of cancer cells in melanoma [79], HCC [80], and glioma [8] and decreases stemness in HCC cells [49,73], lung cancer cells [14], and colorectal cancer cells [74]. You et al. [80] demonstrated that a stiff matrix induces the stemness of HepG2 HCC cells, whereas Schrader et al. and Tian et al. [49,73] suggested that a stiff matrix decreases the stemness of Huh7, Hep3B and MHCC97 HCC cells. Thus, stemness may be differently regulated by stiffness depending on the cell type in HCC. In addition, the optimum matrix stiffness for maintaining stemness was 5 kPa for mammary cancer cells, 25 kPa for colorectal and gastric cancer cells, and 50 kPa for bone osteosarcoma cells [81]. Therefore, the specific stiffness of the matrix in specific cell types may be important for the induction and maintenance of cancer stem cells.

### 3.7. Angiogenesis Regulated by Matrix Stiffness

Angiogenesis is important for the development of tumors and cancer progression because cancer cells can obtain sufficient nutrients and oxygen via a newly generated vascular system [38]. Previous studies have indicated that matrix stiffness is a crucial factor in angiogenesis. Vascular endothelial growth factor (VEGF), a key secretion molecule for angiogenesis, is upregulated in HCC cells and blood vessel endothelial cells on a stiff matrix [82,83], indicating that matrix stiffening causes angiogenesis in tumors.

### 3.8. Avoiding Immune Destruction Regulated by Matrix Stiffness

Avoiding immune destruction is essential for cancer progression to maintain cancer cell overgrowth [38]. The expression of programmed death-ligand 1 (PD-L1) in cancer cells plays an important role in the escape from cell death in cancer cells by the immune system. Previous studies have demonstrated that PD-L1 expression is upregulated by a stiffer matrix in lung cancer cells [5] and mammary cancer cells [84]. These results suggest that cancer cells in a stiff matrix evade immune destruction by expressing PD-L1.

## 4. Matrix Stiffness-Sensitive Transcription Factors Regulate Cancer Progression

Regulation of gene transcription plays a central role in the response to specific stimuli and determines cellular functions. Transcription factors that bind to specific DNA sequences and positively or negatively regulate downstream transcription are key molecules for transcription patterns [85]. Therefore, in mechanotransduction, the conversion of mechanical cues such as stiffness of the matrix to biochemical signaling in the cells, transcription factors play pivotal roles. The important behaviors of transcription factors include nuclear localization, expression and activation. Here, we introduce transcription factors that respond to matrix stiffness and their roles in cancer progression (Table 1).

### 4.1. YAP/TAZ in Epithelial/Cancer Cells

**Figure 2:**
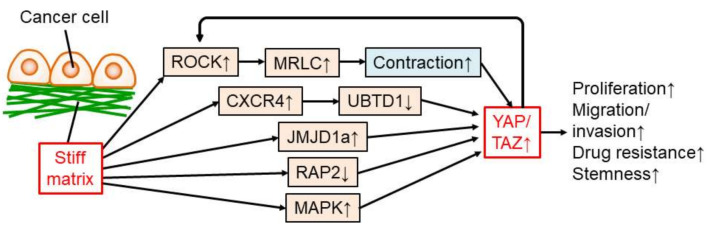
Regulation and function of YAP/TAZ in cancer cells modulated by a stiff matrix. ↑: upregulation, ↓: downregulation.

The Yorkie-homologues Yes-associated protein (YAP) and/or transcriptional coactivator with PDZ-binding motif (TAZ), also known as WWTR1, are the most well-studied transcription factors regulated by matrix stiffness. A stiff matrix induces the nuclear localization and/or expression of YAP/TAZ followed by transcriptional regulation in mammary epithelial/cancer cells [40,41,70,86,87,88], cervical cancer cells [86], pancreatic cancer cells [23,71], colorectal cancer cells [50], lung cancer cells [51,90], hepatocellular carcinoma cells [55,56,91], ovarian cancer cells [58], melanoma cells [92], osteosarcoma cells [93], and prostate cancer cells [90]. In bladder cancer patients, nuclear localization of YAP is higher in recurrent, stiffer cancer tissues than in newly diagnosed, softer cancer tissues [9]. Basal and Her2+ mammary cancer (stiff and more aggressive) patients have stronger YAP staining than luminal (soft and less aggressive) mammary cancers [107]. Furthermore, upregulation of YAP/TAZ by a stiff matrix contributes to cancer progression via proliferation and drug resistance in mammary epithelial/cancer cells [40,70,87], proliferation and EMT in pancreatic cancer cells [23,71], proliferation in colorectal cancer cells [50], growth of lung cancer cells [51], drug resistance, migration, proliferation, EMT, and stemness in HCC cells [55,56,91], and migration and invasion of prostate cancer cells [90]. Therefore, YAP/TAZ upregulation by a stiff matrix is critical for many steps of cancer progression in various types of cancers (Figure 2).

A stiff matrix regulates YAP/TAZ in cancer cells via several molecular mechanisms (Figure 2). One of the dominant mechanisms is the regulation of actomyosin contraction. A stiff matrix induces Rho-kinase (ROCK) signaling, resulting in phosphorylation of myosin regulatory right chain (MRLC) and contractile force in actomyosin, which then translocates YAP/TAZ to the nucleus in cancer cells [50,86,108,109]. In fibroblasts, contraction of the cytoskeleton by a stiff matrix leads to forces exerted through focal adhesions to the nucleus and nuclear flattening, which stretches nuclear pores and increases nuclear import of YAP [110]. In addition, ROCK2 expression is upregulated by active YAP, suggesting a positive feedback loop between ROCK signaling and YAP activation in cancer cells [109]. The stiff matrix promotes the expression of C-X-C motif chemokine receptor 4 (CXCR4) and decreases the level of ubiquitin domain-containing protein 1 (UBTD1), which is involved in the proteasome-dependent degradation of YAP, and finally enhances YAP activity in cancer cells [90,91]. Matrix stiffening triggers upregulation of the histone demethylase Jumonji domain-containing 1A (JMJD1a), leading to YAP/TAZ transcription in carcinoma cells [25]. Ras-related GTPase RAP2 is another important molecule for YAP/TAZ activation—that is, RAP2 is inactivated by a stiff matrix, leading to YAP/TAZ activation in cancer cells [88]. In addition, mitogen-activated protein kinase (MAPK) signaling is activated by a stiffer matrix and activates YAP in the nuclei of HCC cells [56].

The YAP/TAZ regulate genes critical for cancer progression. For instance, YAP prevents cell cycle exit by promoting *Skp2* transcription in mammary epithelial cells and cancer cells [87]. In addition, YAP activity regulates *MMP7* expression and proliferation in colorectal cancer cells [50]. Furthermore, YAP/TAZ play a dominant role in mechano-regulated transcription, as the depletion of YAP/TAZ abolishes the ECM stiffness-responsive transcriptome in HEK293A kidney cells [88]. These results suggest that YAP/TAZ have critical functions in the transcription for malignancy in cancer cells.

Several studies have indicated that YAP/TAZ activity, controlled by matrix stiffness, is dependent on other environmental factors and cell types. In stiff and soft 3D matrixes, MCF10A mammary epithelial cells do not show YAP nuclear localization, whereas on 2D matrix cultures, as many studies demonstrated, MCF10A cells display YAP nuclear localization on a stiff matrix [89]. In contrast, a stiff matrix localizes YAP to the nuclei of melanoma and lung cancer cells in vivo [14,92]. In addition, YAP expression and activity in MDA-MB-231 mammary cancer cells were highest on the 38 kPa matrix than on the 10 or 57 kPa matrices [76]. Thus, the contribution of matrix stiffness to YAP/TAZ activity may be differently regulated by cell type and culture dimension.

### 4.2. YAP/TAZ in Stromal Cells in Cancer

**Figure 3:**
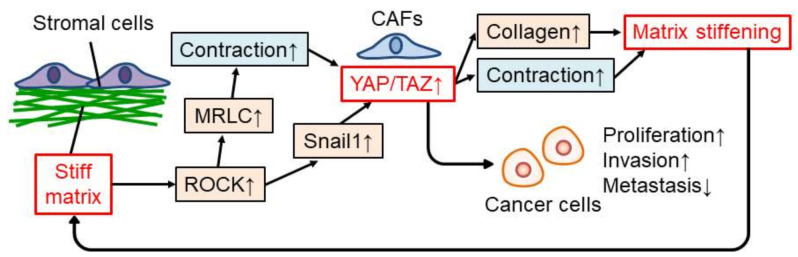
Regulation and function of YAP/TAZ in stromal cells modulated by stiff matrix. ↑: upregulation, ↓: downregulation.

The YAP/TAZ also play a critical role in mechanotransduction in stromal cells in cancer (Figure 3). In stromal cells, matrix stiffness induces collagen production and contraction, which are typical characteristics of CAFs, by activating YAP via ROCK-MRLC-regulated actomyosin contraction [15,27,94]. Collagen production and contraction of CAFs then enhance matrix stiffening; therefore, there is a positive feedback loop between matrix stiffening and stromal cells, especially CAFs [15]. YAP activation in CAFs is also triggered by a stiff matrix by increasing snail protein via ROCK activity [13]. Furthermore, CAFs promote proliferation and the invasion of cancer cells and prevent metastasis [15,27]. In addition, a stiff matrix induces YAP/TAZ-dependent glutamate/aspartate crosstalk between cancer cells and CAFs in tumors, resulting in cancer progression [67]. Therefore, the activity of YAP/TAZ, which is regulated by matrix stiffness in stromal cells, is also crucial for cancer progression.

### 4.3. β-Catenin

The function of β-catenin as a transcription factor in cancer progression is regulated by matrix stiffness. A stiff matrix increased the nuclear accumulation of β-catenin in skin epithelial cells [45], mammary epithelial/cancer cells [95], pancreatic cancer cells [71], HCC cells [96], and glioma cells [8]. Transcriptional activation of β-catenin by a stiff matrix promotes the proliferation of skin epithelial cells [45] and stemness in glioma cells [8]. In HCC cells, a stiff matrix induces osteopontin expression via the integrin β1/GSK-3β/β-catenin signaling pathway and may accelerate HCC progression [96]. Another study showed that a stiff matrix induces cellular communication network factor 1 (CCN1) expression, which induces β-catenin activity and N-cadherin expression on the surface of endothelial cells and promotes endothelial cell–cancer cell interaction to enhance intravasation and metastasis [66]. Therefore, a stiff matrix was suggested to positively regulate cancer progression via β-catenin activity.

### 4.4. NF-κB

There are contradictory reports on nuclear factor kappa-B (NF-κB) activation by matrix stiffness. In lung cancer cells, NF-κB is temporarily localized to nuclei and activated on a stiff matrix via MRLC phosphorylation and induces morphological changes [97]. In contrast, mammary cancer cells have been suggested to increase NF-κB activity on the soft matrix and enhance drug resistance [75]. It has been suggested that NF-κB activation by matrix stiffness is dependent on the cell type.

### 4.5. Twist1

The Twist family BHLH transcription factor 1 (Twist1) is localized to nuclei and activated by a stiff matrix in mammary epithelial cells and triggers EMT, migration, and invasion [41,53]. In addition, Twist1 expression was positively correlated with tumor stiffness in mammary cancer patients [98]. High matrix stiffness promotes the nuclear localization of Twist1 by releasing Twist1 from its cytoplasmic binding partner G3BP2 [53]. Thus, Twist1 functions in mechanotransduction in cancer cells and plays an important role in cancer progression.

### 4.6. HIF1A

Hypoxia-inducible factor 1-alpha (HIF1A) is a transcription factor that responds to multiple stimuli, including hypoxia [111]. The stiff matrix promotes HIF1A expression in glioma cells and, as a result, increases tenascin C expression, which is critical for glioma aggression [7]. In mammary cancer, HIF1A expression is positively correlated with stiffer tissues in mammary cancer patients [98]. Another study showed that tamoxifen reduces HIF1A expression in stromal cells by suppressing myosin-dependent contraction and matrix stiffening in pancreatic cancer [99]. Macrophages are also sensitive to matrix stiffness; a stiff matrix strengthens the polarization of M2 macrophages. HIF1A-dependent LOXL2 expression is triggered by a stiff matrix in M2 polarized macrophages [21]. Thus, HIF1A is also a mechanosensitive transcription factor involved in cancer progression.

### 4.7. Snail

The transcription factor Snail, which is regulated by matrix stiffness, plays an important role in cancer progression. A soft matrix protects mammary epithelial cells from multinucleation, linked to drug resistance and invasion potential, by preventing Snail-induced upregulation of the filament-forming protein septin-6 [100]. A stiff matrix induces Snail expression, and as a result, triggers EMT and metastasis in HCC cells [63]. Snail is also important for stromal cells in tumors. Snail protein levels increase and accumulate in the nuclei of mammary cancer cells and CAFs by stiff matrices in culture and in vivo [13]. Snail is essential for CAFs to express molecules that induce matrix stiffening [13]. A stiff matrix induces ROCK activity, which stabilizes Snail protein and localizes Snail to the nucleus via MAPK signaling [13]. Snail also regulates YAP activity triggered by matrix stiffness in CAFs [13], suggesting crosstalk between transcription factors in stiff tumor microenvironments.

### 4.8. Another Transcription Factors

A stiff matrix induces phosphorylation of Smad2/3 via actomyosin contractions in HCC cells [49,101]. The SRY-box transcription factor 2 (SOX2) is upregulated for the induction of stemness by stiff matrix in HCC cells [80], whereas SOX2 expression is induced by a soft matrix for drug resistance to apoptosis in laryngeal squamous cell carcinoma cells [72], suggesting a cell type-dependent response of SOX2 to matrix stiffness. Phosphorylation of signal transducer and activator of transcription 3 (STAT3) is enhanced by a stiff matrix in pancreatic cancer cells and is associated with shorter patient survival [102]. Activator protein 1 (AP-1) transcriptional activation through the JNK/c-Jun signaling pathway induced by a stiff matrix promotes LOXL2 expression in HCC cells [103]. In lung cancer cells, increased matrix stiffness triggers activation of lymphoid enhancer binding factor 1 (LEF1) and c-Myb transcription factors and increases discoidin domain receptor tyrosine kinase 2 (DDR2) expression for EMT, invasion, and proliferation [104]. A stiff matrix induces nuclear localization of myocardin-related transcription factor A (MRTF-A) in osteosarcoma cells and promotes EMT and migration of osteosarcoma cells [59]. In mammary cancer cells, a stiff matrix increases p53 expression in the nuclei and p53 transcriptional activity after treatment with doxorubicin, then decreases doxorubicin-resistance [105]. A stiff matrix activates RhoA-Akt-p300 mechanotransduction to enhance stromal cell activation and promotes metastasis of colorectal cancer cells to the liver [106]. ZNF217 increases in the normal mammary epithelium of women with high mammographic density and mammary epithelial cells on a 2D or in a 3D stiff matrix, correlating positively with epithelial proliferation and density [44]. High Nanog expression in colorectal cancer cells cultured in a 3D soft matrix induces stemness [74]. These transcription factors are sensitive to matrix stiffness and may play important roles in cancer progression.

## 5. Conclusions

In this review, we discuss matrix stiffening in tumors, which is regulated via cancer and stromal cells. The stiff matrix regulates cancer progression both positively and negatively by modulating proliferation, migration, invasion, metastasis, drug resistance, stemness, angiogenesis and immune response in tumors. These phenomena are widely regulated by transcription factors such as YAP/TAZ, β-catenin, NF-κB, Twist1, HIF1A, and Snail. YAP/TAZ are also critical for tissue stiffening; therefore, a positive feedback loop between YAP/TAZ and the extracellular matrix might be one of the major regulators of cancer progression in solid cancer. Thus, transcription factors such as YAP/TAZ of cancer and stromal cells in tumors are potential therapeutic targets for cancer treatment. However, as shown above, contradictory roles of these factors in cancer progression have been reported. For example, a stiff matrix induces EMT, invasion and metastasis in mammary cancer [53], whereas it prevents metastasis by triggering prosaposin secretion from stromal cells [27]. This may be dependent on the interaction between the matrix and specific cells; in addition, other factors such as cancer type and tissue heterogeneity appear to be critical. In the future, a more precise explanation of the interaction between specific cancer cells, stromal cells, and the extracellular matrix in heterogenous cancer tissues is crucial.

Matrix stiffness is a key factor in the positive and negative regulation of cancer progression by modulating epithelial/cancer cells and stromal cells by stimulating transcription factors. Therefore, matrix stiffness and transcription factors are potential therapeutic targets for cancer treatment. Previous studies have suggested that tamoxifen treatment reduces HIF1A levels by suppressing mechanotransduction in pancreatic cancer [99]. Tamoxifen has also been reported to inhibit YAP activation, HIF1A levels, and synthesis of matrix proteins in stromal cells for matrix stiffening [112]. Future therapies for cancer patients may not only target cancer cells themselves but also control stromal cells and matrix mechanics to prevent cancer progression via multiple steps, including mechanotransduction.

## Figures and Tables

**Table 1:** Transcription factors regulated by matrix stiffness in cancer.

Name of Transcription Factors (TFs)	Cancer/Cell Types	Regulation of TFs by Stiff Matrix	The Results of Upregulated or Downregulated TFs by Stiff Matrix	References
YAP/TAZ	Mammary epithelial/cancer cells	Upregulation	Proliferation↑ Drug resistance↑	[40,41,70,86,87,88]
	Cervical cancer cells	Upregulation	-	[86]
	Mammary epithelial cells	Upregulation on 2D matrix, No significant changes in 3D matrix	-	[89]
	Pancreatic cancer cells	Upregulation	Proliferation↑ EMT↑	[23,71]
	Colorectal cancer cells	Upregulation	Proliferation↑	[50]
	Lung cancer cells	Upregulation	Growth↑	[51,90]
	Hepatocellular carcinoma cells	Upregulation	Drug resistance↑ Migration↑ Proliferation↑ EMT↑ Stemness↑	[55,56,91]
	Ovarian cancer cells	Upregulation	-	[58]
	Melanoma cells	Upregulation	-	[92]
	Osteosarcoma cells	Upregulation	-	[93]
	Prostate cancer cells	Upregulation	Migration, invasion↑	[90]
	Bladder cancer tissues	Upregulation	-	[9]
	Stromal cells/Cancer associated fibroblasts (CAFs)	Upregulation	Matrix remodeling↑ Cancer cell proliferation↑ Cancer cell invasion↑ Metastasis↓ Differentiation to CAFs from stromal cells↑	[13,15,27,94]
β-catenin	Skin epithelial cells	Upregulation	Proliferation↑	[45]
	Mammary epithelial/cancer cells	Upregulation	-	[95]
	Pancreatic cancer cells	Upregulation	-	[71]
	Hepatocellular carcinoma cells	Upregulation	-	[96]
	Glioma cells	Upregulation	Stemness↑	[8]
	Endothelial cells	Upregulation	Intravasation of cancer cells↑	[66]
NF-κB	Lung cancer cells	Upregulation	Morphological changes	[97]
	Mammary cancer cells	Downregulation	Chemo-, radio-resistance↑	[75]
Twist1	Mammary epithelial/cancer cells	Upregulation	EMT↑ Migration, invasion↑	[41,53,98]
HIF1A	Glioma cells	Upregulation	Aggression↑	[7]
	Mammary cancer cells	Upregulation	-	[98]
	Stromal cells in pancreatic cancer	Upregulation	Matrix remodeling↑	[99]
	Macrophages	Upregulation	Matrix remodeling↑	[21]
Snail	Mammary epithelial cells	Upregulation	Multinucleation↑	[100]
	Hepatocellular carcinoma cells	Upregulation	EMT↑ metastasis↑	[63]
	Cancer associated fibroblasts	Upregulation	Matrix remodeling↑	[13]
Smad2/3	Hepatocellular carcinoma cells	Upregulation	-	[49,101]
SOX2	Hepatocellular carcinoma cells	Upregulation	Stemness↑	[80]
	Laryngeal squamous cell carcinoma cells	Downregulation	Drug resistance↓	[72]
STAT3	Pancreatic cancer cells	Upregulation	-	[102]
AP-1	Hepatocellular carcinoma cells	Upregulation	Matrix remodeling↑	[103]
c-Myb, LEF1	Lung cancer cells	Upregulation	EMT↑ Invasion↑ Proliferation↑	[104]
MRTF-A	Osteosarcoma cells	Upregulation	EMT↑ Migration↑	[59]
p53	Mammary cancer cells	Upregulation	Drug resistance↓	[105]
p300	Stromal cells in liver	Upregulation	Myofibroblast activation↑ Metastasis↑	[106]
ZNF217	Mammary epithelial cells	Upregulation	Proliferation↑	[44]
Nanog	Colorectal cancer cells	Downregulation	Stemness↓	[74]

↑: upregulation, ↓: downregulation.
